# Gabapentin for complex regional pain syndrome in Machado-Joseph disease: a case report

**DOI:** 10.1186/1752-1947-5-268

**Published:** 2011-07-01

**Authors:** Yang-Ching Lo, Kwong-Kum Liao, Yi-Chung Lee, Bing-Wen Soong

**Affiliations:** 1 Department of Neurology, Taipei Veterans General Hospital, No. 201, Section 2, Shih-Pai Road, Beitou District, Taipei, Taiwan, 112

## Abstract

**Introduction:**

Chronic pain is a common problem for patients with Machado-Joseph disease. Most of the chronic pain in Machado-Joseph disease has been reported to be of musculoskeletal origin, but now there seems to be different chronic pain in patients with Machado-Joseph disease.

**Case presentation:**

A 29-year-old man (Han Chinese, Hoklo) with Machado-Joseph disease experienced severe chronic pain in both feet, cutaneous thermal change, thermal hypersensitivity, focal edema, and sweating and had a history of bone fracture. These symptoms were compatible with a diagnosis of complex regional pain syndrome. After common analgesics failed to relieve his pain, gabapentin was added and titrated to 2000 mg/day (500 mg every six hours) in less than two weeks. This relieved 40% of his pain and led to significant clinical improvement.

**Conclusions:**

The pathophysiology of complex regional pain syndrome includes peripheral and central sensitizations, the latter of which might be associated with the neurodegeneration in Machado-Joseph disease. In this report, we suggest that gabapentin could inhibit central sensitization as an adjunct for complex regional pain syndrome in patients with Machado-Joseph disease.

## Introduction

Machado-Joseph disease (MJD), also called spinocerebellar ataxia type 3, is the most common subtype of spinocerebellar ataxias worldwide and is caused by a CAG trinucleotide repeat expansion in the coding region of the *MJD1 *gene. The main features of MJD are ataxia and ophthalmoparesis and pyramidal, extrapyramidal, and amyotrophic signs [1]. Chronic pain is one of the most disabling symptoms of MJD. Nearly 80% of the chronic pain in MJD has been reported to be of musculoskeletal origin [2]. To the best of our knowledge, no previous case report has mentioned complex regional pain syndrome (CRPS) in patients with MJD. In the case reported here, a patient with MJD experienced symptoms of CRPS, which were relieved by gabapentin.

### Case presentation

A 29-year-old man (Han Chinese, Hoklo) with a diagnosis of MJD and CAG repeat numbers of 14 and 70 in the *MJD1 *gene had been confined to a wheelchair for three years. He had a history of left humeral bone fracture as a result of an accidental fall one year earlier. Three months prior to his stay in the hospital, he began to have severe regional pain in his feet. His pain was spontaneous, continuing, and excruciating in the areas mentioned above. He described it as like "originating deeply from the bone" and denied any burning or lancinating sensation. Moderate hair loss, focal edema, cutaneous thermal change with hypersensitivity to cold temperatures, and intermittent sweating in both feet were observed. Yet there was no light-touch allodynia, fever, chill, or local tenderness. He could hardly do anything except moan in bed in the daytime and yell and kick almost every night.

**Figure 1:**
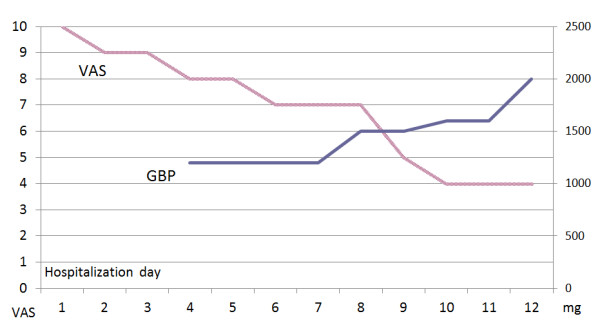
**Visual analogue scale (VAS) and daily dosage of gabapentin (GBP) in the hospital**. Our patient's pain was gradually resolved from 8 to 4 on the VAS by an increase in GBP from 1200 to 2000 mg. VAS and GBP values are presented on the left y-axis and right y-axis, respectively.

Plain film of both feet revealed normal alignment and no bone lesions. A nerve conduction study and electromyography revealed merely mild sensorimotor polyneuropathy. Quantitative sensory testing (QST) was conducted for his severely depressed mood. During his stay in the hospital, we used the visual analogue scale (VAS) (0 for no pain and 10 for maximal pain) to measure his pain intensity [3]. Initially, his pain was minimally relieved, from 10 to 8 on the VAS, by common analgesics, including acetaminophen, diclofenac, and tramadol. On day four, gabapentin was added at a daily dose of 1200 mg (400 mg every eight hours) (Figure 1). With an increasing daily dosage of gabapentin from 1200 to 2000 mg (500 mg every six hours), his pain was gradually resolved from 8 to 4 on the VAS (Figure 1) by day 10. The abnormal cutaneous thermal change and edema also disappeared. As his pain diminished significantly, we observed a remarkable improvement in his quality of life: he slept better, was more mobile, and had more daily activity. Finally, he could move around again by wheelchair.

## Discussion

In this case, all of the clinical features of pain-such as spontaneous continuing regional pain with cutaneous thermal change, thermal hypersensitivity, focal edema, moderate hair loss as trophic change, sweating, immobility, and history of bone fracture-implied CRPS [4]. CRPS is characterized by a continuing (spontaneous or evoked) regional pain that is seemingly disproportionate in time or degree to any known trauma or lesion. In the latest proposed criteria for CRPS, regional pain is not limited to a nerve territory or dermatome, which is usually accompanied by a distal predominance of abnormal sensory, motor, sudomotor, vasomotor, and trophic changes [5]. Mechanical or thermal hypersensitivity is the hallmark of nociceptive pain compatible with CRPS [6,7].

In the pathophysiology of CRPS, peripheral nerve or soft tissue injuries trigger the initial peripheral nociceptive sensitization caused by prostanoids, kinins, and cytokines. Afterwards, the calcium influx and activity-dependent plasticity alter the pain transmission neurons, which undergo central sensitization and lead to a major physiological change of the autonomic, pain, and motor systems [8]. Given the small-fiber distal axonopathy that is possibly a cause of CRPS [9], QST should be managed for the evaluation of small-fiber functions with cold and heat pain thresholds [10].

To the best of our knowledge, no large clinical trials in the pharmacologic treatment for CRPS have been conducted. Most of the pharmacologic rationales for CRPS (such as topical agents, anti-epileptic drugs, tricyclic anti-depressants, and opioids) were applied by the treatment of other neuropathic pain syndromes, which are strongly related to gabapentin [11]. It has been reported that gabapentin at daily doses of 900 to 2400 mg [12,13] could be considered to control CRPS, but the effects seemed to be limited. We suppose that, since the neurodegeneration in MJD is multi-systemic (involving the somatosensory cortex), gabapentin might play a role in central neuromodulation via the alpha-2-delta subunit of the voltage-gated calcium channels [14]. This successful experience suggests that the adjunctive effect of gabapentin is due to inhibition at the central sensitization.

## Conclusions

Our experience offers a new option to patients with MJD and chronic pain. The exact mechanism remains to be elucidated; however, gabapentin could be considered an adjunct for the treatment of CRPS in patients with MJD.

## Abbreviations

CRPS: complex regional pain syndrome; MJD: Machado-Joseph disease; QST: quantitative sensory testing; VAS: visual analogue scale.

## Consent

Written informed consent was obtained from the patient for publication of this case report and any accompanying images. A copy of the written consent is available for review by the Editor-in-Chief of this journal.

## Competing interests

The authors declare that they have no competing interests.

## Authors' contributions

YCLo was responsible for the treatment to relieve our patient's CRPS and was a major contributor in writing the manuscript. KKL interpreted the possible pain mechanism in MJD and edited the manuscript. YCLe performed genotyping and genetic diagnosis for our patient. BWS offered the knowledge of his research in spinocerebellar ataxia. All authors read and approved the final manuscript.
